# Assessment of the Effects of Dietary Vitamin D Levels on Olanzapine-Induced Metabolic Side Effects: Focus on the Endocannabinoidome-Gut Microbiome Axis

**DOI:** 10.3390/ijms222212361

**Published:** 2021-11-16

**Authors:** Armita Abolghasemi, Claudia Manca, Fabio A. Iannotti, Melissa Shen, Nadine Leblanc, Sébastien Lacroix, Cyril Martin, Nicolas Flamand, Vincenzo Di Marzo, Cristoforo Silvestri

**Affiliations:** 1 Centre de Recherche, l’Institut Universitaire de Cardiologie et de Pneumologie de Québec (CRIUCPQ), Québec, QC G1V 4G5, Canada; armita.abolghasemi@usherbrooke.ca (A.A.); claudia.manca.1@ulaval.ca (C.M.); melissa.shen.1@ulaval.ca (M.S.); nadine.leblanc@fsaa.ulaval.ca (N.L.); sebastien.lacroix.8@ulaval.ca (S.L.); cyril.martin@criucpq.ulaval.ca (C.M.); nicolas.flamand@criucpq.ulaval.ca (N.F.); vincenzo.dimarzo@criucpq.ulaval.ca (V.D.M.); 2 Département de Médecine, Faculté de Médecine, Université Laval, Québec, QC G1V 0A6, Canada; 3 Joint International Unit between the National Research Council (CNR) of Italy and Université Laval on Chemical and Biomolecular Research on the Microbiome and Its Impact on Metabolic Health and Nutrition (UMI-MicroMeNu), Université Laval, Québec, QC G1V 0A6, Canada; 4 Joint International Unit between the National Research Council (CNR) of Italy and Université Laval on Chemical and Biomolecular Research on the Microbiome and Its Impact on Metabolic Health and Nutrition (UMI-MicroMeNu), Institute of Biomolecular Chemistry, National Council of Research (Consiglio Nazionale delle Ricerche), 80087 Pozzuoli, Italy; fabio.iannotti@icb.cnr.it; 5 Institut sur la Nutrition et les Aliments Fonctionnels (INAF), Québec, QC G1V 0A6, Canada; 6 École de Nutrition, Faculté des Sciences de L’agriculture et de L’alimentation (FSAA), Université Laval, Québec, QC G1V 0A6, Canada; 7 Centre Nutrition, Santé et Société (NUTRISS), Université Laval, Québec, QC G1V 0A6, Canada

**Keywords:** endocannabinoidome, endocannabinoids, gut microbiome, vitamin D, olanzapine, antipsychotic, obesity, lipidomics

## Abstract

Vitamin D deficiency is associated with poor mental health and dysmetabolism. Several metabolic abnormalities are associated with psychotic diseases, which can be compounded by atypical antipsychotics that induce weight gain and insulin resistance. These side-effects may be affected by vitamin D levels. The gut microbiota and endocannabinoidome (eCBome) are significant regulators of both metabolism and mental health, but their role in the development of atypical antipsychotic drug metabolic side-effects and their interaction with vitamin D status is unknown. We studied the effects of different combinations of vitamin D levels and atypical antipsychotic drug (olanzapine) exposure on whole-body metabolism and the eCBome-gut microbiota axis in female C57BL/6J mice under a high fat/high sucrose (HFHS) diet in an attempt to identify a link between the latter and the different metabolic outputs induced by the treatments. Olanzapine exerted a protective effect against diet-induced obesity and insulin resistance, largely independent of dietary vitamin D status. These changes were concomitant with olanzapine-mediated decreases in *Trpv1* expression and increases in the levels of its agonists, including various *N*-acylethanolamines and 2-monoacylglycerols, which are consistent with the observed improvement in adiposity and metabolic status. Furthermore, while global gut bacteria community architecture was not altered by olanzapine, we identified changes in the relative abundances of various commensal bacterial families. Taken together, changes of eCBome and gut microbiota families under our experimental conditions might contribute to olanzapine and vitamin D-mediated inhibition of weight gain in mice on a HFHS diet.

## 1. Introduction

Atypical antipsychotic drugs, including quetiapine, clozapine, olanzapine and ziprasidone, are used to treat psychotic disorders, especially the acute phase of schizophrenia. These drugs, compared to typical antipsychotics, exhibit wider receptor affinity profiles as antagonists and greater affinity for serotonin-2 compared with D2 dopamine receptors [1,2,3]. Atypical antipsychotics offer several notable benefits over typical ones, such as improved cognitive function and prevention of deterioration of the quality of life; however, they also cause a variety of side effects including obesity, insulin resistance and diabetes as well as dyslipidemia, which in turn are associated with long-term cardiovascular health risks [4]. This is particularly troubling given that the psychological conditions for which these drugs are prescribed show significant association with overweight and obesity in connection with poor diets, including increased intake of refined carbohydrates and fat [5]. The gut microbiome plays a role in the dysfunction of the metabolic cycle induced by olanzapine. A study in rats suggested that changes in the fecal microbiota profile of olanzapine-treated rats might be related to the dysmetabolic effects of this drug [6], while germ-free mice are largely resistant to olanzapine-mediated weight gain, which is reversed when gut microbiota have been reintroduced [7]. In addition, studies in rats suggest that vitamin D deficiency affects metabolic disorders caused by antipsychotic drugs [8].

Although vitamin D is mostly produced endogenously from exposure to UV rays from sunlight, and can be obtained through diet, vitamin D deficiency is widespread [9,10]. Vitamin D deficiency has been proposed to contribute to the dysregulation of insulin production and sensitivity and adipose tissue function [11,12], and to be associated with mental illnesses, including schizophrenia [13]. Extreme vitamin D deficiency is prevalent in the acute state of schizophrenia, which may be the result of low sunlight exposure, intake of antipsychotic medications, poor mobility, bad dietary habits, excessive alcohol intake, and/or smoking, all features of psychotic patients [14,15]. A clinical trial with a combination of vitamin D and probiotics for 12 weeks in schizophrenic patients showed clinical improvements in their psychotic symptoms and metabolic profile [16].

Dysbiosis is defined as an imbalance between protective and harmful bacterial species lining the intestine and known as the gut microbiota that may harm the host [17]. Gut microorganisms modify the host metabolic balance and are an important factor for the development of some conditions associated with the metabolic syndrome [18], including insulin resistance and fatty liver [19]. Vitamin D deficient diets change the gut microbiota taxa by increasing the relative abundance of the *Enterobacteriaceae, Prevotella* and *Actinomyces,* and decreasing *Odoribacteraceae*, although they do not cause generalized dysbiosis [20], thus affecting food digestion and energy metabolism [21,22].

Several studies have shown the existence of a functional interaction between the brain and commensal bacteria in the gut, known as the microbiota–gut–brain axis, affecting behavior and its disturbances, including anxiety and depression [23,24]. A role in this axis is played by the endocannabinoidome (eCBome), which refers to the endocannabinoid lipid mediators, anandamide (AEA) and 2-arachidonoylglycerol (2-AG), plus several other families of related endocannabinoid-like long chain fatty acid derivatives, such as the AEA and 2-AG congeners, the *N*-acylethanolamines (NAEs) and 2-mono-acylglycerols (2-MAGs), respectively. The tissue levels of these mediators are regulated by a host anabolic and catabolic enzymes as well as by the dietary intake of the corresponding fatty acids, and their biological actions are mediated by a plethora of receptors beyond the canonical cannabinoid receptor 1 and 2 (CB1; *CNR1* and CB2; *CNR2*) [25]. There is an upregulation of endocannabinoid activity at CB1 receptors in abdominally obese compared to lean individuals [26]. The eCBome appears to have a bi-directional interaction with gut microbiota which has important consequences on the regulation of metabolism [27,28,29,30,31]. Germ-free mice exhibit significant alterations not only within the intestinal, but also the brain eCBome, reverted at least in part by faecal microbiota transfer from conventional donor mice [32], whereas altering gut microbial composition through the use of prebiotics modifies CNR1 expression in obese mice [33]. Further, treatment with *A*. *muciniphila*, a metabolically beneficial commensal intestinal bacterium, increased the levels of 2-MAGs, including 2-AG, 2-Oleoylglycerol (2-OG) and 2-Palmitoylglycerol (2-PG), in the colon of mice with diet-induced obesity [34], and 2-PG levels in the plasma of obese individuals [35].

In the present study, we examined whether changes in dietary vitamin D are implicated in the progression and severity of metabolic abnormalities associated with olanzapine use in a high-fat, high-sucrose (HFHS) model of diet-induced obesity (DIO) in mice, since obesity and higher refined carbohydrate and fat consumption is associated with psychotic disorders. We also examined the gut microbiota-eCBome axis in these mice as we hypothesized that it may play a role in mediating the metabolic effects of antipsychotics and their potential modulation by vitamin D.

## 2. Results

### 2.1. Olanzapine Reduces HFHS Diet-Mediated Weight Gain and Adiposity in Female Mice

**Figure 1:**
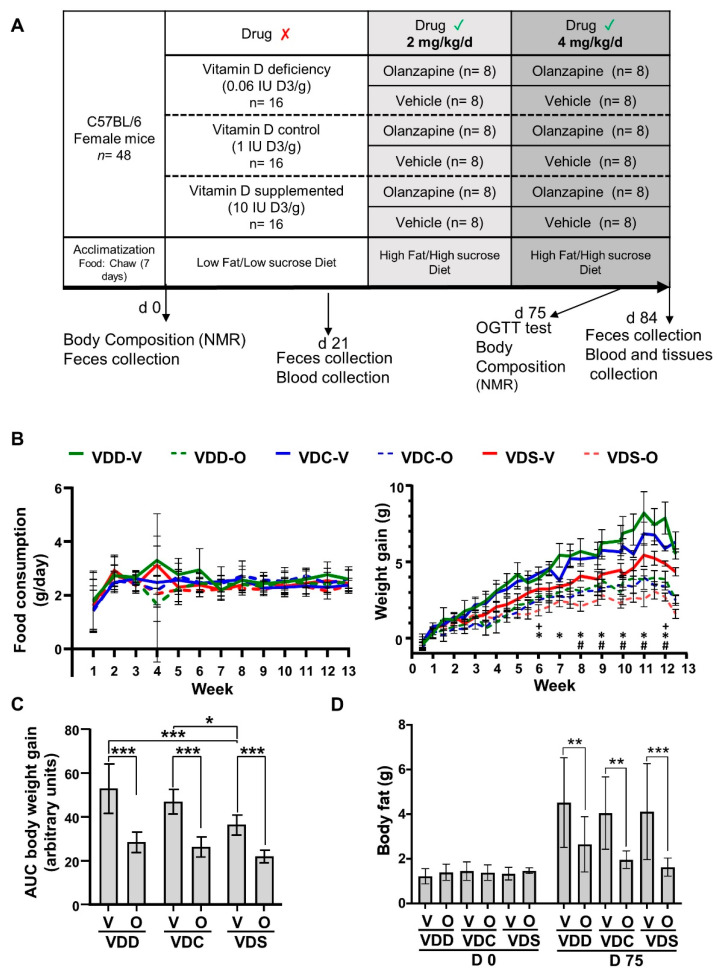
(**A**) Study design. Effect of vitamin D and olanzapine on (**B**) food consumption (**left**) and body weight **(right**; +, *, # *p* ≤ 0.05 for VDD-O vs. VDD-V, VDC-O vs. VDC-V, VDS-O vs. VDS-V, respectively), (**C**) AUC of body weight gain and (**D**) body fat mass of mice treated with olanzapine or vehicle on day 0 and day 77. * *p* ≤ 0.05; ** *p* ≤ 0.01; *** *p* ≤ 0.005. All data are shown as mean ± SD, *n* = 8 per group). VDD, Vitamin D Deficiency; VDC, Vitamin D Control; VDS, Vitamin D supplemented; O, Olanzapine; V, Vehicle.

We first determined the effect of olanzapine on diet-induced obesity (DIO) and associated metabolic complications utilizing a HFHS diet, and if olanzapine-induced effects could be modified by previous and concomitant exposure to varying levels of dietary vitamin D. Neither the vitamin D status nor olanzapine treatment was found to have an effect on HFHS food consumption (Figure 1B, left). The vitamin D status of the vehicle groups (i.e., VDD-V, VDC-V and VDS-V) did not statistically significantly alter weight gain (Figure 1B, right). However, assessment of body weight gain by area under the curve (AUC) analysis for these groups showed that the VDD-V and VDC-V groups had significantly greater AUCs than the VDS-V group, suggesting that vitamin D supplementation stunted HFHS-induced DIO (Figure 1C). Interestingly, despite the lack of change in food intake in olanzapine-treated groups (i.e., VDD-O, VDC-O and VDS-O) as compared to vehicle controls, body weight gain presented significant decreases in all groups with olanzapine exposure (average final differences in weight at end of the study: VDD-V vs. VDD-O; 1.53 g, VDC-V vs. VDC-O 2.05 g, VDS-V vs. VDS-V; 1.80 g [*p* ≤ 0.05 for all comparisons], Figure 1B). While for the VDD-O group this was only evident at week 6 and 12, while for the VDC-O and VDS-O groups this was consistent from week 6 and 8, respectively. The AUC analysis for body weight gain supported this latter finding, with olanzapine treatment resulting in lower AUCs regardless of the vitamin D content of the diet (Figure 1C). Body composition analysis determined by NMR revealed that neither dietary vitamin D levels nor olanzapine treatment modified lean mass by day 77 of the study (Supplementary Figure S1), but that fat mass was significantly lower in all olanzapine treated groups as compared to their respective controls (Figure 1D).

**Figure 2:**
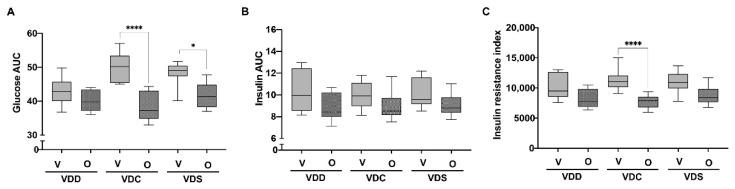
Area under the curve (AUC) analysis obtained from OGTTs for (**A**) glucose and (**B**) insulin and (**C**) insulin resistance index which is calculated by multiplying the AUC of glucose and insulin. Data are shown as a box (5th and 95th and mean value) and whisker (minimum and maximum values) plots (*n* = 8 per group). * *p* ≤ 0.05; **** *p* ≤ 0.005. VDD, Vitamin D Deficiency; VDC, Vitamin D Control; VDS, Vitamin D supplemented; O, Olanzapine; V, Vehicle.

In order to determine if the lower weight gain observed in mice treated with olanzapine resulted in improved glucose handling, mice were subjected to an oral glucose tolerance test (OGTT) at day 79. AUC analysis of blood glucose levels measured over 2 h after a dextrose bolus found that VDC-O and VDS-O, but not VDD-O, groups had lower values than their vehicle controls (Figure 2A), while a similar AUC analysis of insulin levels showed no differences between groups (Figure 2B), suggesting that in the former two groups insulin sensitivity was increased. However, the VDC-O group was the only one found to have a significantly reduced insulin resistance index (*p* = 0.0016; Figure 2C).

### 2.2. Olanzapine Modifies Several Aspects of Adipose Tissue eCBome Signaling

**Figure 3:**
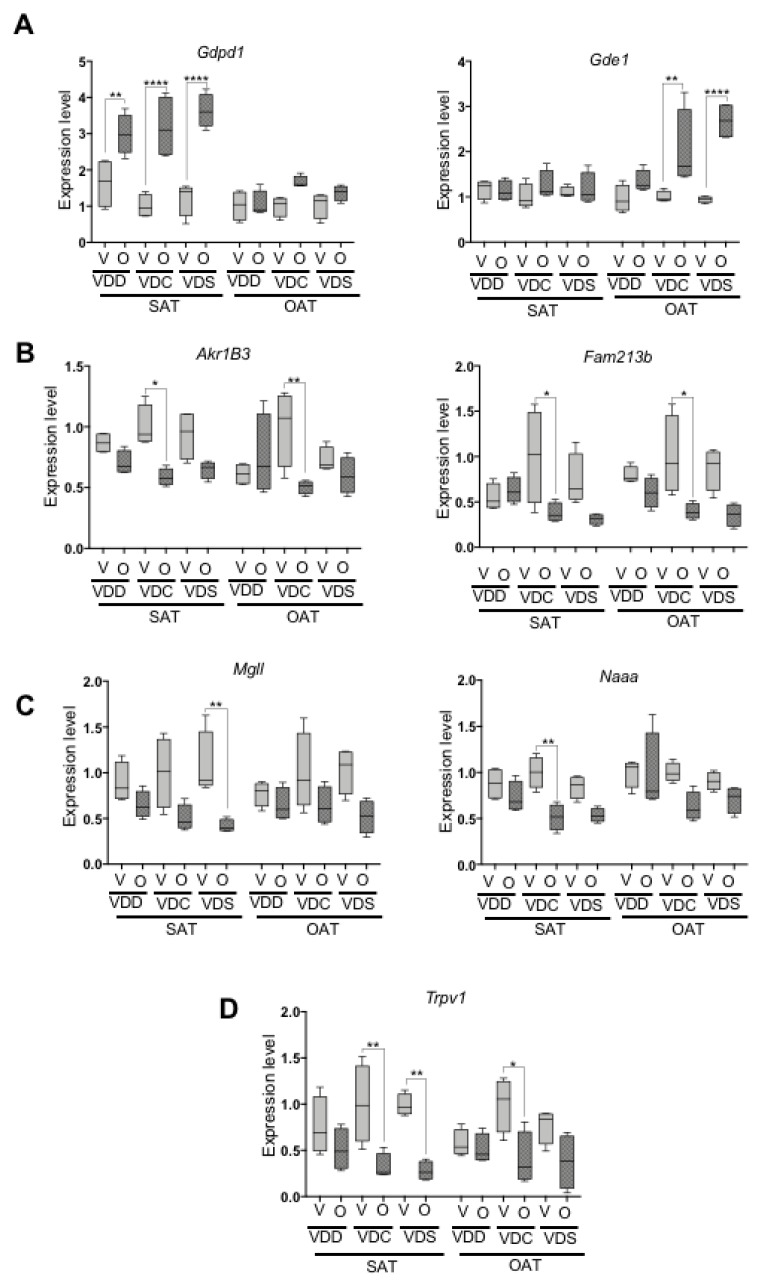
mRNA expression levels of eCBome related genes measured by qPCR, including (**A**) anabolic enzymes involved in NAE biosynthesis, (**B**) anabolic enzymes involved in synthesizing prostaglandins and prostaglandin-related lipids from fatty acids or endocannabinoidome (eCBome) mediators, respectively, (**C**) catabolic enzymes and (**D**) one selected receptor. Data are shown as a box (5th and 95th and mean value) and whisker (minimum and maximum values) plots. * *p* ≤ 0.05; ** *p* ≤ 0.01; **** *p* ≤ 0.001. VDD, Vitamin D Deficiency; VDC, Vitamin D Control; VDS, Vitamin D supplemented; O, Olanzapine; V, Vehicle; SAT, Subcutaneous adipose tissue; OAT, Ovarian adipose tissue.

We next went on to determine if olanzapine modified the eCBome within the subcutaneous (SAT) and ovarian (OAT) adipose tissues. qPCR analysis was performed utilizing a targeted array of 52 eCBome gene receptors and catabolic and anabolic enzymes and four housekeeping genes [32]. The complete list of genes detected within the adipose tissues and their relative expression compared to VDC-C revealed that olanzapine modified the expression of several eCBome genes, often in an adipose depot-specific manner (Supplementary Figure S2). *Gdpd1* and *Gde1*, which encode for enzymes that are involved in *N*-acylethanolamine (NAE) biosynthesis, showed increased expression in olanzapine-treated groups independent of dietary vitamin D levels in a depot-specific (Figure 3A). In SAT, *Gdpd1* expression was significantly increased in olanzapine-treated groups under all diets with different vitamin D levels, while no changes were observed in OAT. Instead, *Gde1* expression was not altered in SAT, while olanzapine increased *Gde1* expression significantly in VDC and VDS diets.

Interestingly, the expression of two genes (*Akr1b3* and *Fam213b*) that encode for enzymes capable of synthesizing PGF2-alpha and PGF2aEA from arachidonic acid and AEA, respectively, were similarly modified by olanzapine. Both these genes showed decreased expression in both SAT and OAT, though only in a statistically significant manner in mice on the VDC diet (Figure 3B), with a strong trend towards a decrease for *Fam213b* in the OAT in mice on the VDS diet (*p* = 0.055).

The major regulator of 2-MAG degradation and final enzymatic step of lipolysis towards the generation of free fatty acids and glycerol, MGLL, was similarly found to generally have decreased gene expression in olanzapine-treated groups (Figure 3C), significantly so within the VDS group in SAT. Similarly, *N*-acylethanolamine acid amide hydrolase (NAAA) expression, which encodes for *N*-acylethanolamine acid amidase, a major degrader of saturated and monounsaturated *N*-acylethanolamines, was also generally down-regulated in VDC-O and VDS-O groups in both adipose tissue depots, with significant decreases specifically observed within the VDC-O in SAT (Figure 3C).

The only eCBome receptor for which we observed a change in expression was *Trpv1*, with olanzapine significantly decreasing *Trpv1* expression in the SAT of mice on the VDC and VDS diets and in the OAT of mice on the VDC diet (Figure 3D).

**Figure 4:**
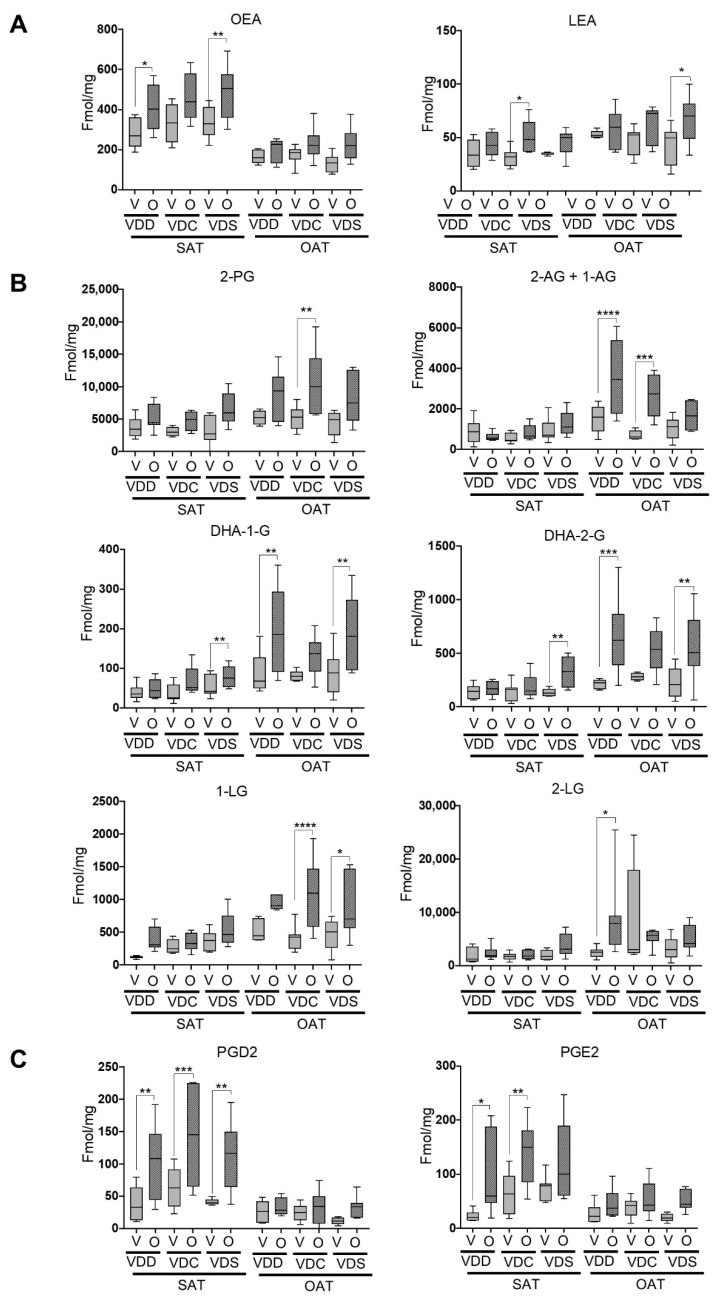
Concentrations of eCBome selected congeners in subcutaneous (SAT) and ovarian (OAT) adipose tissues. Levels of (**A**) NAEs, (**B**) 2-MAGs and (**C**) prostaglandins are expressed as pmol/mg of tissue. Data are shown as box (5th and 95th and mean value) and whisker (minimum and maximum values) plots. * *p* ≤ 0.05; ** *p* ≤ 0.01; *** *p* ≤ 0.005; **** *p* ≤ 0.001. VDD, Vitamin D deficiency; VDC, Vitamin D control; VDS, Vitamin D supplemented; O, Olanzapine; V, Vehicle; SAT, Subcutaneous adipose tissue; OAT, Ovarian adipose tissue.

We next went on to measure the levels of eCBome lipid mediator levels within the SAT and OAT. Interestingly, all the lipids that we analyzed (see Supplementary Figure S3 for a complete list) whose levels were responsive to olanzapine treatment were found to be increased. Two NAEs, i.e., oleoylethanolamide (OEA) and linoleoylethanolamide (LEA), both of which are TRPV1 and GPR119 agonists, were increased in the olanzapine groups, though significant results were more broadly observed in SAT than OAT (Figure 4A). Conversely, while 2-MAG levels showed similar increases in olanzapine-administered groups, where they were found to be more often significantly increased in the OAT than the SAT (Figure 4B). The classic endocannabinoid 2-arachidonoyl glycerol (2-AG and 1-AG) and its congeners 2-palmitoyl glycerol (2-PG) and 2-linoleoyl glycerol (2-LG and 1-LG) were upregulated by olanzapine exclusively in the OAT. Interestingly, increased levels of dietary vitamin D blunted olanzapine-mediated increases in 2-AG and 1-AG, rendering the increase statistically insignificant under the VDS diet. The omega-3 polyunsaturated fatty acid-containing 2-MAG, 2-docosahexaenoyl glycerol (2-DHAG and 1-DHAG), while similarly affected by olanzapine in OAT as the 2-MAGs mentioned above, was also significantly increased in SAT, but only in VDS-O compared to VDS-V.

Finally, we found that olanzapine increased the levels of the prostaglandins PGE_2_ and PGD_2_ in the SAT, without modifying them in the OAT, and that this effect appeared to be largely independent of dietary vitamin D levels (Figure 4C).

### 2.3. Vitamin D Status and Olanzapine Effects on the Gut Microbiome of Mice on High-Fat, High-Sucrose Diets

We next attempted to determine if the vitamin D status and/or olanzapine treatment modified the fecal microbiota of female mice on an HFHS diet. Twenty-one days of the VDD, VDC and VDS LFLS diets did not result in global composition differences in the gut microbiomes as determined by PCoA analysis coupled with PERMANOVA (Supplementary Figure S4A; left). Relative abundance analysis suggested that the proportion of various families were altered however (Supplementary Figure S4A; right). Post-hoc analysis of the relative abundance of the most prevalent bacterial taxa (at least 1%) showed some changes in the family level under these diets before the switch to the HFHS diets. At the family level *Clostridiales vadinBB60* was increased while *Peptococcaceae* was decreased, while the genera *Lachnoclostridium* (belonging to *Lachnospiraceae*), *Butyricicoccus* and *Anaerotruncus* (belonging to *Clostridaceae*) were increased in response to increasing dietary vitamin D levels (Supplementary Figure S5). These effects were lost, however, after 65 days on the HFHS diets, under which no taxa were found to be responsive to vitamin D (data not shown).

**Figure 5:**
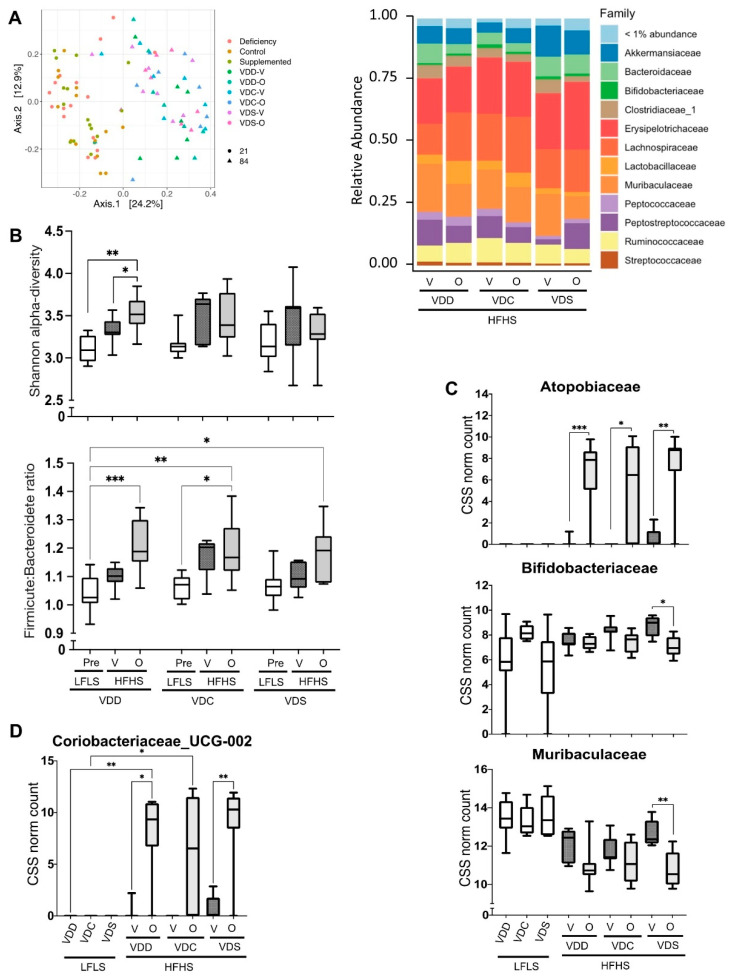
Composition of the faecal microbiome. (**A**) beta-diversity of gut microbiome composition through Principal Coordinate Analysis (PCoA; **left**) and relative abundance of Families (**right**). (**B**) Shannon alpha-diversity index evaluating gut microbiota richness and evenness (**top**) and Firmicutes to Bacteroidetes ratio (**bottom**). Prevalence of selected gut microbiota at the (**C**) family and (**D**) genus level on days 21 (after LFLS) and 86 (after HFHS). Data are shown as box (5th and 95th and mean value) and whisker (minimum and maximum values) plots. * *p* ≤ 0.05; ** *p* ≤ 0.01;, *** *p* ≤ 0.005.

After 65 days on the various HFHS diets, evident shifts within the microbiomes were induced, regardless of the vitamin D status (Figure 5A, left). As with the LFLS diets, vitamin D status had no discernable global effects on the gut microbiomes in mice fed the HFHS diets (Supplementary Figure S4B). Interestingly, olanzapine induced global changes in the gut microbiomes but only under the VDD diet (Supplementary Figure S4C). These effects on the microbiome are also evident when assessing Shannon alpha diversity; vitamin D status had no effect under LFLS or HFHS vehicle diets, but olanzapine treatment of the VDD group significantly increased Shannon alpha diversity when comparing day 21 (end of LFLS diet) to day 86 (end of HFHS diet) in the VDD-O group, as well as when comparing VDD-V to VDD-O after 65 days on the HFHS (Figure 5B, top). Similarly, dietary vitamin D did not alter the Firmicutes/Bacteroidetes ratio under either the LFLS or HFHS diets, however, after 65 days on HFD, it was significantly increased in the VDD-O and VDC-O groups (Figure 5B, bottom).

Differential abundance testing indicated that olanzapine altered the abundance of certain families under the HFHS diet (Figure 5A, right). Statistical analysis of individual taxa identified several olanzapine-responsive bacterial families (olanzapine effect; *p* < 0.05): *Rhizobiaceae, Eggerthellaceae, Bacteroidaceae, Streptococcaceae, Clostridiaceae_1, Enterobacteriaceae* and *Bacteroidaceae*, however post-hoc analysis found that only *Atopobiaceae, Muribaculaceae* and *Bifidobacteriaceae* abundances were altered with respect to vehicle controls (Figure 5C). While *Atopobiaceae* abundance increased with olanzapine under all HFHS diets irrespective of vitamin D levels, *Muribaculaceae* and *Bifidobacteriaceae* were decreased, and only in a statistically significant manner in the VDS group (Figure 5C). At the genus level, only one taxon was found to be significantly modified by olanzapine in post-hoc analyses; *Coriobacteriaceae*_UCG-002 was barely detectable in faeces of vehicle controls, but increased significantly in response to olanzapine, independent of the vitamin D dietary status (Figure 5D).

## 3. Discussion

Obesity is associated with different parameters such as vitamin D deficiency, psychiatric disorders, dysbiosis of gut microbiota and dysregulation of endocannabinoid and eCBome signaling. Vitamin D deficiency is associated with a wide variety of diseases including psychotic disorders such as schizophrenia in addition to obesity and the metabolic syndrome [36,37,38]. Vitamin D supplementation in healthy overweight and obese women increased vitamin D blood concentrations and led to body fat mass reduction [39]. A study on female schizophrenic patients being treated with olanzapine found that administration of vitamin D was generally effective at reducing obesity [40], whereas, conversely, schizophrenic patients with vitamin D deficiency who were under treatment with clozapine did not undergo changes in their metabolic profile nor psychotic status [41].

Olanzapine is an effective second-generation/atypical antipsychotic that produces less adverse effects for movement including akathisia, dystonia and hypertonia, although it tends to cause more weight gain than typical antipsychotics [42]. The use of atypical antipsychotic drugs is correlated with a major risk of metabolic side effects, leading to dyslipidemia and obesity. Several studies have shown that female rats [6,43,44] and mice [45,46] display metabolic changes similar to the side-effects observed in patients taking olanzapine. These murine studies have also found that olanzapine induces changes in and that its metabolic effects are dependent on, the gut microbiome [6,47] and the endocannabinoid system [48]. Most recently, it was also demonstrated that olanzapine pharmacokinetics can be modified by changes within the microbiome [49], further demonstrating the interaction of this drug with commensal microbes. Based on the above, we hypothesized that vitamin D could modify the severity of olanzapine-induced metabolic side effects, with increased food intake and weight gain in response to olanzapine in vitamin D deficiency and less weight gain in response to olanzapine with vitamin D supplementation as compared to control levels of vitamin D. Surprisingly, the results of our study showed a significant decrease in weight gain and body fat mass and decreased insulin resistance in olanzapine-treated groups compared to their controls. On the other hand, less surprisingly, increasing vitamin D levels showed trends for a reduction in body weight gain and fat mass in both vehicle and olanzapine treated groups, confirming the effect of vitamin D on obesity. However, no such trend was observed for insulin resistance.

Insulin has many physiological activities including reduction of blood glucose and stimulation of fatty acid synthesis, processes that become dysregulated in obesity due to hyperinsulinemia and the development of insulin resistance [50].We report here that the DIO-induced glucose dysmetabolism was surprisingly improved by chronic olanzapine treatment, with the VDC-O and VDS-O groups showing significantly improved glucose levels in an OGTT, while the VDC-O group also had an insulin resistance index. Interestingly, regardless of the levels of dietary vitamin D, all groups showed trends in for improvement in these measures. These effects are different from those reported by Townsend et al. [51], who found that mice on a high fat diet have exacerbated acute olanzapine-induced glucose dysmetabolism. Considering the interaction between body fat mass and insulin resistance, our results suggest that under our HFHS diet, the increased insulin sensitivity in response to olanzapine is in part the result of olanzapine-induced reduction in fat mass and weight gain, a condition which could not have been obtained in the acute treatment mentioned above.

Although the effects of olanzapine reported here are surprising considering previous human and rodent studies with this drug, several differences in our protocol might explain this finding. Unlike most other studies, olanzapine was administered here concomitantly with a HFHS diet and subsequently to a priming LFLS diet period with lower or higher dietary vitamin D levels. However, our results show that the drug was effective at reducing body weight and fat mass also in mice under a normal vitamin D dietary regimen. However, a previous study with female CD1 mice showed that 4 weeks of 8 mg/kg, but not 4 mg/kg, olanzapine administered using a subcutaneous osmotic minipump did produce an enhancement of body weight and fat mass [52]. Additionally, in C57BL/6J female mice oral administration of olanzapine (3 mg/kg), doubled weight gain under a high-fat diet (40% energy from fat) without affecting weight gain in a control diet (8% energy from fat) [53]. Somewhat in agreement with this, Cope et al. treated female C57BL/6J with either a low or high fat diet (4.6 vs. 15.6% fat by weight) for 3 weeks and found that olanzapine (9 mg/kg orally) induced similar weight gain regardless of diet, as was observed in mice treated with either risperidone or quetiapine [54]. In rats fed an HFHS diet (45 kcal% fat, 25 kcal% sucrose), 10 mg/kg of olanzapine over 6 weeks resulted in significantly more weight gain than vehicle, but in chow-fed rats olanzapine inhibited weight gain [55]. In contrast, the same group previously found that nether clozapine or quetiapine potentiated HFHS-induced obesity, or induced weight gain on their own, in the same experimental model used in their olanzapine study [56]. However, intraperitoneal treatment of female rats on a high fat diet (35% kcal from fat) with either olanzapine (2 mg/kg), risperidone (0.5 mg/kg) or ziprasidone (2.5 mg/kg) found that none of the treatments resulted in significant weight gain above controls, and the olanzapine group showed a tendency for a decrease in weight gain [57]. Thus, a combination of different routes of administration and doses of olanzapine, different species/strain and sex, presence and composition of concomitant HFHS diet might explain our surprising finding of beneficial metabolic effects of olanzapine.

The eCBome, a complex lipid signaling system including endocannabinoids, other fatty acid derived mediators, their receptors and anabolic and catabolic enzymes, is involved in the control of energy metabolism and body weight [58]. The importance of the endocannabinoid system role in body weight control is exemplified by data from *Cnr*^−/−^ (CB1^−/−^) mice, which have significantly decreased caloric intake and body weight compared to control mice [59,60,61]. Indeed, CB1 receptors appear to play a significant role in mediating some olanzapine metabolic side effects. Olanzapine-treated rats exhibited weight gain and increased blood glucose, increased biosynthetic eCBome enzyme expression, but not *Cnr1* expression, in the nucleus accumbens, and decreased expression of peroxisome proliferator-activated receptors PPARA (a nuclear hormone receptor that is regulated by multiple NAEs and 2-MAGs) in the liver, compared to control rats [48]. Antagonism of CB1 receptors decreased *Cnr1* mRNA expression and reversed olanzapine-induced changes in eCBome enzymes and PPARA expression and metabolic side effects [48].

In partial agreement with these previous data, we found here that olanzapine treatment was accompanied by changes in the white adipose tissue (WAT) expression of eCBome metabolic enzymes that might underlie the observed alteration in eCBome mediator levels. Accordingly, while the NAE biosynthetic enzymes were up-regulated by the drug, 2-MAG and NAE catabolic enzymes were down-regulated, thus possibly explaining why we found a general elevation of both NAEs and 2-MAGs in the WAT of olanzapine-treated female mice. Since *Mgll* encodes for the enzyme catalysing the last and rate-limiting step of lipolysis, by hydrolysing MAGs to fatty acids and glycerol [62,63], the lower expression levels of this gene induced by olanzapine may also explain why olanzapine-treated mice had less fat mass independently of the observed higher 2-MAG levels, similar to *Mgll* knockout mice under a high fat diet [30]. At any rate, none of the NAEs and 2-MAGs that we found to be elevated by olanzapine are capable of activating CB1, and hence producing dysmetabolic effects in the context of obesity, with the exception of the endocannabinoid 2-AG. In fact, all the eCBome mediators elevated by olanzapine are capable, through different receptors (TRPV1, PPARs, GPR119 or CB2), to produce metabolically beneficial and/or anti-inflammatory effects and may therefore underlie part of the effects observed with olanzapine [31]. The elevation of 2-AG would be in line with a role for CB1 signaling in olanzapine-mediated dysmetabolism, which has been shown to be reversed in rats treated with various CB1 antagonists [48]. While this would be consistent with increased in 2-AG in the visceral adipose tissue of patients with obesity [64], they are not consistent with our observation that under a HFHS-diet olanzapine decreased weight gain and adiposity. Furthermore, in rats on a chow diet, CB1 receptor occupancy is was reported to be increased in various regions of the brain, including the hypothalamus, which was reduced under the conditions of a high fat diet [65]. The significance of this is unclear however, as the authors reported no olanzapine induced weight gain under either diet. Further, our own analysis of the hypothalamus found no changes in CB1 gene expression (data not shown).

In particular, TRPV1 channels, expression of which either showed strong trends for or significantly decreased expression in all olanzapine-treated groups in SAT and OAT, are modulated by several eCBome mediators, including long-chain-saturated NAEs, MAGs, *N*-acyldopamines, and *N*-acyltaurines [58]. Several NAEs, including AEA, OEA, LEA and PEA, have been shown to activate TRPV1 [60,63]. Additionally, different MAGs including 1-monoacylglycerols (1-MGs) having C18 and C20 unsaturated and C8–C12 saturated fatty acid, and 2-MAGs having C18 and C20 unsaturated fatty acids, activate TRPV1 receptors [66,67]. Administration of dietary capsaicin, an exogenous agonist of TRPV1, to mice fed a high fat diet resulted in lower fasting glucose, insulin and leptin levels, and improved glucose tolerance [68]. Activation of TRPV1 through capsaicin promotes energy metabolism and suppresses visceral fat accumulation. MAGs with unsaturated long-chain fatty acid increase the expression of UCP-1 and decrease the weight of epididymal white adipose tissue, serum glucose, total cholesterol and free fatty acid levels in male mice under a high fat-high sucrose diet [69]. However, it has been shown that *Trpv1*^−/−^ mice on high-fat diet have lower body weight compared to wild type mice [70]. Thus, it is possible that both TRPV1 activation and desensitization, which immediately follows channel activation, may produce beneficial metabolic effects. Indeed, increasing the levels of NAEs including PEA and OEA is accompanied by TRPV1 desensitization [71], and PEA was suggested to be more effective at desensitizing this channel than capsaicin [72]. It has also been shown that white adipocyte-restricted *Napepld* knockout in mice leads to reduced PEA and OEA levels in the WAT, with concurrent downregulation of *Ucp1* expression and WAT browning [73]. Treatment with probiotics reverted both effects, suggesting a role of these NAEs in WAT browning. The results from our study show that olanzapine decreases the expression of *Trpv1* and increases the WAT levels of its NAE and 2-MAG agonists, which may cause overall activation and desensitization of TRPV1, with weight loss and decreased fat mass, possibly through reduction in adipogenesis and lipogenesis and stimulation of WAT browning.

Vitamin D modifies PGE_2_ levels directly in different cell types [74,75], however, we did not see any changes in PGE_2_ or PGD_2_ levels between groups under different vitamin D diets. Previous studies have demonstrated administration of olanzapine reduces PGE_2_ levels in different parts of the brain in vivo and in vitro [76,77]. While we did not observe any such changes in the hypothalamus (data not shown), we found an enhancement of PGE_2_ and PGD_2_ levels, particularly in the SAT. Prostaglandins, including PGD_2_ and PGE_2_, are synthesized from arachidonic acid and are involved in the differentiation, maturation and function of white adipocytes [78] PGE_2_ has a key role in the browning of the WAT [79], while conversely inhibiting the activity of hormone-sensitive lipase (HSL), the most important lipolytic enzyme in WAT, resulting in an anti-lipolytic action in obese WAT [80]. Lipolysis stimulation increased the release of PGE_2_ and PGD_2_, and enhanced COX-2 expression in obese WAT cells in vitro. PGE_2_ is also suggested to mediate macrophage migration into adipose tissue but without inducing inflammation during weight loss while PGD_2_ appears to have less of an effect [81]. However, PGD_2_ enhances adipogenesis and lipid accumulation in adipocytes, presumably by preventing lipolysis [73,82]. Therefore, the decreased fat mass accumulation in our experimental mice belonging to the olanzapine treatment groups may be linked also to increased levels of PGE_2_ within the WAT, which may result in the stimulation of pathways inducing browning of adipocytes, modifying lipid metabolism and inhibiting adipogenesis, whereas the concomitant olanzapine-mediated increase in PGD_2_ might be expected to counteract this, thus making the functional role of prostaglandins in mediating the metabolic effects described here an open question that merits further investigation.

The gut microbiome has a crucial role in energy regulation and obesity. A major part of human gut bacteria belongs to three phyla; *Firmicutes*, *Bacteroidetes* and *Actinobacteria* [83]. Increasing the proportion of *Firmicutes* and *Actinobacteria* and decreasing that of *Bacteroidetes* may contribute to a higher risk of developing obesity and its metabolic consequences [84,85]. It is possible that some treatments, by altering gut microbiota composition, could ameliorate olanzapine-induced weight gain [6]. Vitamin D regulates the gut microbiome, and its deficiency can result in gut dysbiosis [86,87]. In addition, germ free mice have lower levels of vitamin D [88], which shows an interplay with the gut microbiota. Our results indicate that, while chronic olanzapine under a HFHS diet did not appear to induce global changes in gut bacterial community structure, it did significantly alter the levels of *Atopobiaceae* (increased), *Muribaculaceae* (decreased) and *Bifidobacteriaceae* (decreased), the latter two of which were only modulated under conditions of vitamin D supplementation. A study on mice on a high fat diet feeding demonstrated decreased levels of the *Muribaculaceae* family, which belong to *Bacteriodetes* phylum, in common with significant weight gain in mice [89]. Here we show that olanzapine decreased this family, though only significantly so when administered in conjunction with vitamin D supplementation, suggesting that this little described taxon may also be obesogenic. The relative abundance of *Actinobacteria* is higher in obese people [84]. Although *Bifidobacteriaceae* belong to *Actinobacteria*, this family is known as a generally protective in that it is associated with reduced intestinal inflammation and amelioration of insulin resistance and glucose tolerance [90,91], even though the relative abundances of *Bifidobacteriaceae* increase in diet-induced obesity [92], in agreement with our present finding of olanzapine decreasing both this family and body weight. In a correlation analysis between gut microbiota family and metabolic biomarkers, the *Atopobiaceae* family (also belonging to the *Actinobacteria* phylum) was found to have a negative relationship with triglycerides (TG) and fasting blood glucose [93], which may be seen as in partial agreement with our present finding of olanzapine increasing the relative abundance of this family and the beneficial metabolic effects observed here with this antipsychotic drug. Therefore, as with the eCBome, we have identified olanzapine-induced modifications in the microbiome that correlate with the surprising anti-obesity effects of olanzapine in mice on a chronic HFHS diet.

## 4. Materials and Methods

### 4.1. Animals and Housing

All animal experiments were approved by the Université Laval committee for the protection of animals (license #2018-109). Forty-eight six-week-old, female C57BL/6J mice were purchased from Jackson Laboratory (Bar Harbor, ME, USA) and maintained in the animal facility of the Institut Universitaire de Cardiologie et Pneumologie de Québec (IUCPQ, Québec, QC, Canada). All animals were housed one mouse per cage under a 12 h:12 h light dark cycle with ad libitum access to water. After 7 days of acclimatization, the animals were divided into 3 groups and fed a low-fat low-sucrose purified diet (10% fat and 7% sucrose, D12450J; LFLS) (day 0; start of protocol) for 21 days (*n* = 16 each) with: 1— normal levels of vitamin D (1 IU D3/g body weight) as controls (VDC), 2—low levels of vitamin D (0.06 IU D3/g body weight) to induce deficiencies (VDD) and 3—high levels of vitamin D (10 IU D3/g body weight; supplemented VDS). After 21 days, the LFLS diet was changed to a high-fat, high-sucrose purified diet (45% fat and 17% sucrose, D07081501; HFHS) with the same vitamin D levels and each group was sub-divided into either: 1-vehicle, or 2-olanzapine (*n* = 8 each) for a further 65 days. All diets were produced by Research Diets Inc. (New Brunswick, NJ, USA; Provided by Cedarlane, Burlington, ON, Canada). Detailed diet compositions are provided in Supplementary Table S1.

### 4.2. Drug Administration

Olanzapine was administered by daily gavage in the morning at a dosage of 2 mg/kg/day for the first 35 days and then 4 mg/kg/day for another 28 days.

Olanzapine (Zyprexa, Eli Lilly, Toronto, ON, Canada) (dissolving 5 and 10 mg tablets) was purchased as available on the market. Tablets were dissolved in 1 mL of 0.1 N citric acid, and then adjusted to pH 5.5 with 0.9% sterile PBS, respectively, for the dosage of 2 and 4 mg/kg/day, while the vehicle was prepared using the same proportion of 0.1 N citric acid and PBS [52]. Solutions were prepared freshly before each administration.

### 4.3. Body Composition and Food Intake

In each experimental period, food intake was measured, and food was changed twice a week. Food intake was obtained by subtracting remaining food, including any spilled food in cages, from a weighed aliquot that was initially provided. Weight monitoring was performed twice a week in the morning, before each animal was orally administered olanzapine. Body composition of live animals was determined using the NMR Analyser Minispec (Bruker Optik, GmbH, Rheinstetten, Germany) at days 0 and 77 of the protocol.

### 4.4. Sample Collection

Faeces were collected before 12:00 on day 21 (i.e., start of HFHS) and 84. Briefly, individual mice were placed sterile cages without bedding and resulting faeces were collected within 30 min, immediately frozen on dry ice and then stored in −80 °C.

Blood was collected by standard cardiac puncture method on the day of dissection (day 86) into the 1.3 mL K3 EDTA sampling tube (Sarstedt, Montreal, QC, Canada), blood samples were taken from animals during deep isoflurane anesthesia, after which animals were cervically dislocated and the necessary tissues and organs including different adipose tissues (brown, ovarian and inguinal) were collected.

Tissues were divided into two parts: one for fatty acid and endocannabinoidome lipid measurement by LCMS, which were snap-froze immediately in liquid nitrogen and then kept at −80 °C, and the other part placed in the tubes containing RNAlater™ (Qiagen, Mississauga, Ontario, Canada) for RNA extraction, incubated overnight at 4 °C and stored at −20 °C.

### 4.5. Oral Glucose Tolerance Test (OGTT)

On day 79 of the protocol, all the animals were fasted in new cages with water for 6 h from 7 a.m. to 1 p.m., at which an OGTT was performed. Each mouse was weighed, had a baseline blood glucose measurement performed (Time 0) in blood obtained from the tail vein and then was immediately gavaged with a 50% dextrose solution at a dose of 2 mg/g. Subsequent blood glucose levels were measured at 15, 30, 60, 90 and 120 min from the same tail vein puncture. Blood samples were collected using Microvette^®^ CB300 blood tubes containing EDTA (Sarstedt, Montreal, QC, Canada) at time points of 0, 15, 30, 60 and 120 min for insulin measurement by Elisa (Mouse Ultrasensitive Insulin ELISA kit; Alpco, Salem, NH, USA) according to the manufacturer’s instructions. Glucose levels were quantified using an Accu-Chek^®^ Aviva (Roche Diagnostics, Laval, Quebec) blood glucose meter with Accu-Chek^®^ Aviva test strips (Roche Diagnostics, Quebec, Canada).

The insulin resistance index, which was determined by multiplying the area under the curve of both blood glucose (0 to 120 min) and plasma insulin (0 and 120 min), were obtained following the oral glucose tolerance test.

### 4.6. RNA Isolation and qPCR-Based TaqMan Open Array

RNA was extracted from ovarian and inguinal adipose tissues samples with the RNeasy Lipid Tissue Mini Kit (Qiagen, Mississauga, ON, Canada) according to the manufacturer’s instructions and eluted in 30 μL of UltraPure Distilled Water (Invitrogen, Carlsbad, CA, USA). The concentration and purity of RNA were determined by measuring the absorbance of the RNA in a Biodrop at 260 nm and 280 nm. One microgram of the total RNA was reverse transcribed using the iScript cDNA synthesis kit (Bio-Rad, Hercules, CA, USA) according to the manufacturer’s instructions.

52 eCBome-related genes and 4 housekeeping genes of *Gapdh*, *Hprt*, *Tbp* and *Rps13* were evaluated using a custom designed qPCR-based TaqMan Open Array on a QuantStudio 12K Flex Real-Time PCR System (Thermo Fisher Scientific, San Jose, CA, USA) following the manufacturer’s instruction as described previously [32]. Gene expression levels were evaluated by the 2^ΔΔCt^ method and represented as fold increase with respect to baseline within each tissue.

### 4.7. eCBome Lipid Mediator Analysis

Lipids were extracted from tissue samples according to the Bligh and Dyer method [94]. About 10 mg of each tissue were homogenized in 1 mL of Tris-HCl 50 mM pH 7 and following 1 mL of methanol using a pestle. 5 μL of eCBome deuterated standards and 5.75 μL of 0.1 M acetic acid were added to each tube and then 1 mL of chloroform was added to each sample, vortexed and centrifuged at 3000× *g* for 5 min. The chloroform step was repeated two more times for a total addition of 3 mL of chloroform. The lipid phases were collected and dried by stream of nitrogen and then diluted in 50 μL of mobile phase containing 50% of solvent A (water + 1 mM ammonium acetate + 0.05% acetic acid) and 50% of solvent B (acetonitrile:water 95:5 + 1 mM ammonium acetate + 0.05% acetic acid). Finally, diluted samples were injected onto an HPLC column (Kinetex C8, 150 × 2.1 mm, 2.6 μm, Phenomenex; Torrance, CA, USA) and eluted at a flow rate of 400 μL/min using a discontinuous gradient of solvent A and solvent B [95]. Afterwards, eCBome-related mediators were quantified by HPLC system interfaced with the electrospray source of a Shimadzu 8050 triple quadrupole mass spectrometer and using multiple reaction monitoring in positive ion mode for the compounds and their deuterated homologs.

### 4.8. 16S rRNA Gene Sequencing

DNA was extracted from faeces using the Qiagen DNeasy PowerSoil Pro Kit (Qiagen, Hilden, Germany) according to the manufacturers’ instructions and diluted in 50 μL of UltraPure Distilled Water (Invitrogen, Carlsbad, CA, USA). The concentrations of extracted DNAs were measured by the Quant-iT PicoGreen dsDNA Kit (Thermo Fisher Scientific, Waltham, MA, USA) and the DNAs were stored at −20 °C, until 16S rDNA library preparation.

For the library preparation, the QIAseq 16S Region Panel protocol in conjunction with the QIAseq 16S/ITS 384-Index I (Sets A, B, C, D) kit (Qiagen, Hilden, Germany) were used for amplification and indexing of the V3-V4 region of the 16S rRNA gene for DNA samples. The 16S metagenomic libraries were qualified by Agilent High Sensitivity DNA Kit (Agilent, Palo Alto, CA, USA) using a Bioanalyser to verify the amplicon size (expected size ~600 bp) and quantified with a Qubit (Thermo Fisher Scientific, Waltham, MA, USA). Libraries were then normalized and pooled to 2 nM, denatured and diluted to a final concentration of 10 pM and supplemented with 5% PhiX control (Illumina, San Diego, CA, USA). Sequencing was performed using the MiSeq Reagent Kit V3 (Illumina, San Diego, CA, USA) by an Illumina MiSeq System (Illumina, San Diego, CA, USA).

Detected sequences were processed using the Dada2 package (Version 1.10.1) [35] and taxonomic assignation was performed against the SILVA 132 rRNA reference database [96]. Sequences detected in less than 5% of all samples were filtered out and relative microbiota abundances were obtained by Cumulative Sum Scaling (CSS, MetagenomeSeq R package, version 1.36.0) [97]. The phyloseq R package (version 1.22.3) was used to calculate Shannon alpha-diversity indexes and to compute Bray–Curtis dissimilarity indexes [98]. All 16S sequencing data has been deposited in the NCBI with SRA accession number SUB10439652.

### 4.9. Statistical Analysis

Area under the curve (AUC) analysis was performed with the AUC function of GraphPad Prism (GraphPad Software, version 9, San Diego, CA, USA) utilizing all time points collected during the analyzed experiments (see relevant protocols within the Materials and Methods). Mixed linear regressions (nlme R package, version 3.1-153) followed by Tukey HSD post hoc test or Kruskal–Wallis test followed by Dunn’s multiple comparison tests, for parametric and non-parametric data, respectively, were used to identify significant vitamin D status and/or olanzapine treatment effects on gut microbiota composition and eCBome levels. Spearman correlations were used to investigate associations between microbiota composition and eCBome mediators. Adjustments for multiple testing were obtained using False Discovery Rate (FDR). All results were considered statistically significant at *p* < 0.05 or FDR-adjusted *p* < 0.1. Analyses were performed using both R and GraphPad Prism.

## 5. Conclusions

In conclusion, the results of this study suggest that under HFHS diet consumption, olanzapine may have a protective effect by ameliorating body metabolism with concomitant beneficial changes in eCBome signaling and gut microbiota composition, thus resulting in decreased body fat mass. In particular, these observations are consistent with our findings that olanzapine decreases *Trpv1* expression and increases the levels of its agonists, including NAEs and 2-MAGs. These changes were identified in association with alterations of various commensal microbial family levels. Additionally, vitamin D supplementation consistently showed trends for an improved metabolic status of the mice, although the changes were generally not statistically significant. Taken together, changes of eCBome and gut microbiota families under our experimental conditions might contribute to olanzapine and vitamin D-mediated inhibition of weight gain in mice on a HFHS diet.

## Data Availability

All 16S sequencing data has been deposited in the NCBI with SRA accession number of SUB10439652.

## Supplementary Materials

The following are available online at https://www.mdpi.com/article/10.3390/ijms222212361/s1.
