# Supplementary material for: Assessment of the Effects of Dietary Vitamin D Levels on Olanzapine-Induced Metabolic Side Effects: Focus on the Endocannabinoidome-Gut Microbiome Axis
Source: Int J Mol Sci. 2021 Nov 16;22(22):12361. doi: 10.3390/ijms222212361 (PMC8620071; doi:10.3390/ijms222212361)

Supplemental Table S1: Diet composition

Diet	LFLS						HFHS					
	VDC		VDD		VDS		VDC		VDD		VDS	
	[1.0 IU/g Vitamin D]		[0.1 IU/g Vitamin D]		[10 IU/g Vitamin D]		[1.0 IU/g Vitamin D]		[0.1 IU/g Vitamin D]		[10 IU/g Vitamin D]	
	**gm(%)**	**kcal(%)**	**gm(%)**	**kcal(%)**	**gm(%)**	**kcal(%)**	**gm(%)**	**kcal(%)**	**gm(%)**	**kcal(%)**	**gm(%)**	**kcal(%)**
Protein	19	20	19	20	19	20	24	20	24	20	24	20
Carbohydrate	67	70	66	69	67	70	41	35	40	34	41	35
Fat	2	10	2	10	2	10	3	45	3	45	3	45
**Total**		**100**		**99**		**100**		**100**		**99**		**100**
**kcal/gm**	**3.85**		**3.85**		**3.84**		**4.73**			**4.73**		
**Ingredient**	**gm**	**kcal**	**gm**	**kcal**	**gm**	**kcal**	**gm**	**kcal**	**gm**	**kcal**	**gm**	**kcal**
Casein, 80 Mesh	200	800	200	800	200	800	200	800	200	800	200	800
L-Cystine	3	12	3	12	3	12	3	12	3	12	3	12
Corn Starch	506.2	2025	506.2	2025	506.2	2025	72.8	291	72.8	291	72.8	291
Maltodextrin 10	125	500	125	500	125	500	100	400	100	400	100	400
Sucrose	68.8	275	68.8	275	68.8	275	172.8	691	172.8	691	172.8	691
Cellulose, BW200	50	0	50	0	50	0	50	0	50	0	50	0
Soybean Oil	25	225	25	225	25	225	25	225	25	225	25	225
Lard	0	0	0	0	0	0	0	0	0	0	0	0
Palm Oil	20	180	20	180	20	180	177.5	1598	177.5	1598	177.5	1598
Mineral Mix S10026	10	0	10	0	10	0	10	0	10	0	10	0
DiCalcium Phosphate	13	0	13	0	13	0	13	0	13	0	13	0
Calcium Carbonate	5.5	0	5.5	0	5.5	0	5.5	0	5.5	0	5.5	0
Potassium Citrate, 1 H2O	16.5	0	16.5	0	16.5	0	16.5	0	16.5	0	16.5	0
Vitamin Mix V10001	10	40	0	0	10	40	10	40	0	0	10	40
Vitamin Mix V13201 (w/o Vit D)	0	0	10	40	0	0	0	0	10	40	0	0
Vitamin D, 100,000 IU/gm	0	0	0	0	0.095	0	0.0004	0	0	0	0.0993	0
Choline Bitartrate	0	0	2	0	2	0	2	0	2	0	2	0
**Total**	**1055.05**	**4057**	**1055.05**	**4057**	**1055.15**	**4057**	**858.15**	**4057**	**858.15**	**4057**	**858.24**	**4057**
Vitamin D (IU/4057 kcal)	1060		60		10560		1100		60		10990	
Vitamin D (IU/g diet)*	1		0.06		10.01		1.28		0.07		12.81	

gr(%); % of macronutrient by weight of diet

kcal(%); % of macronutrient contribution by kcal of diet

FigureS1: Body composition

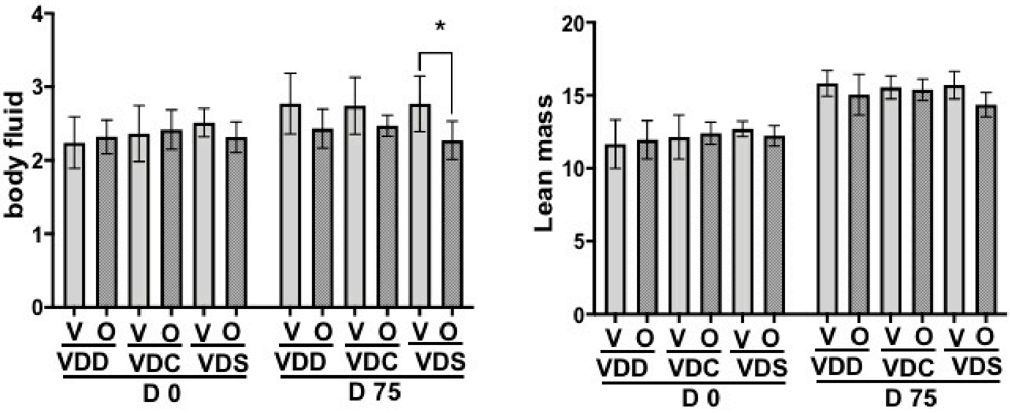

FigureS2: Relative expression of eCBome genes as compared to VDC-V; Blue boxes shows the significant difference between olanzapine and vehicle in each vitamin D level; * shows significancy ($p<0.05$) between different Vitamin D levels, # shows significancy ($p<0.05$) under the effect of both Vitamin D and Olanzapine.

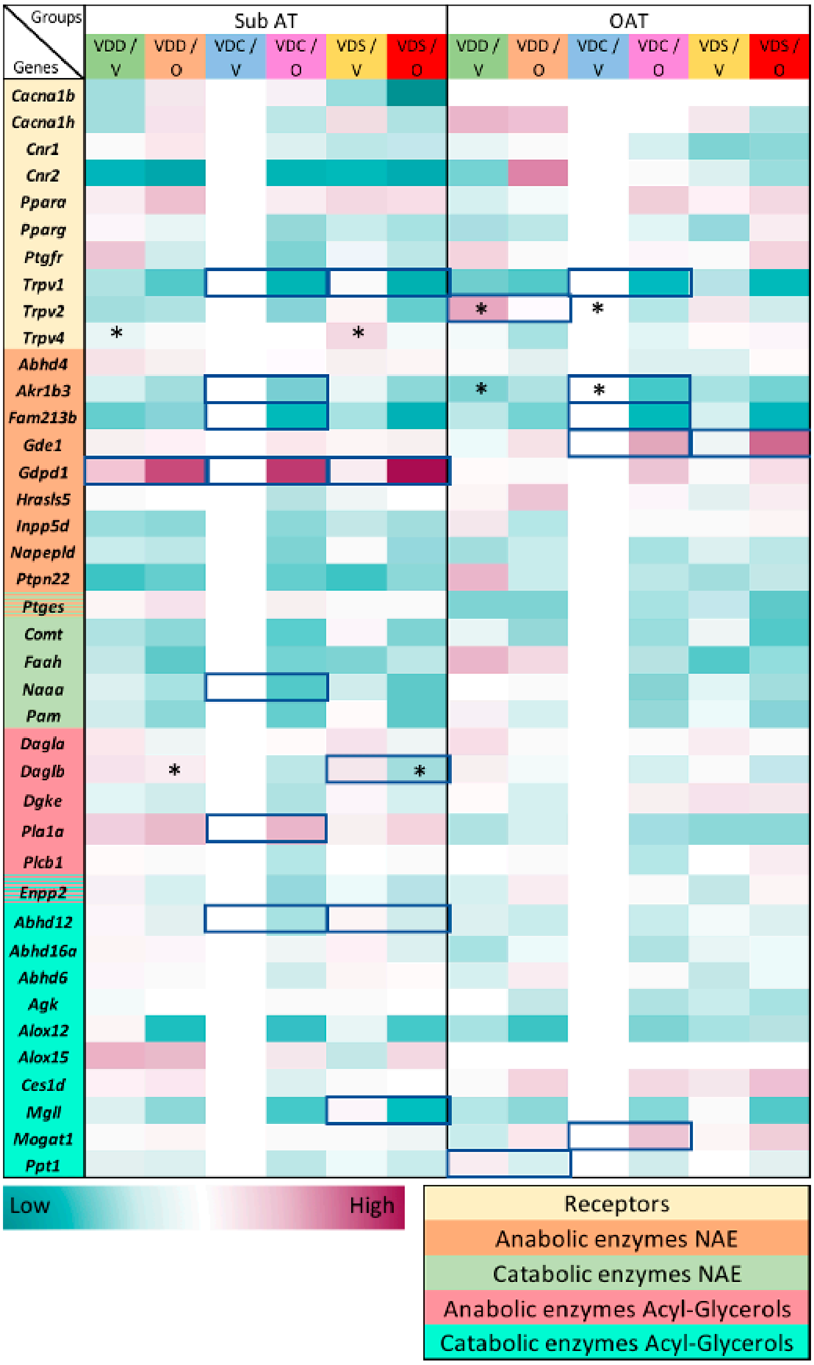

FigureS3: quantification of eCBome mediator levels; Blue boxes show the significant difference between olanzapine and vehicle in each vitamin D level; * shows significancy (p<0.05) between different Vitamin D levels.

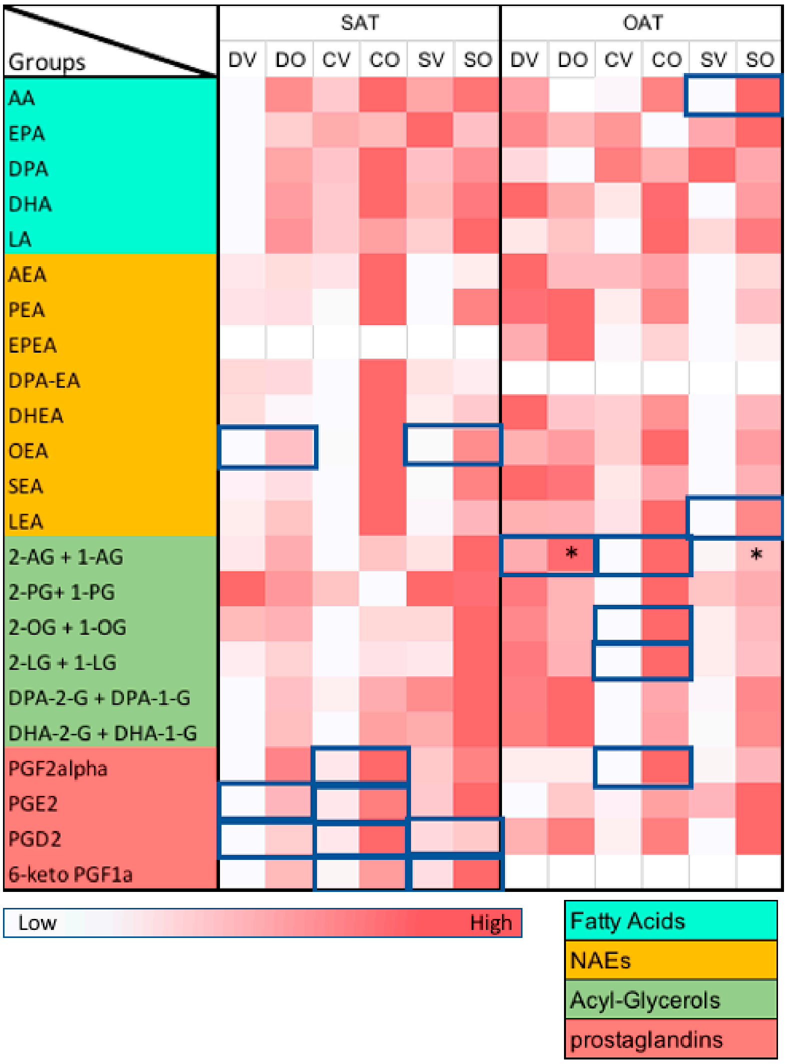

FigureS4: Gut microbiomes composition determined by PCoA coupled with PERMANOVA and differential abundance analysis

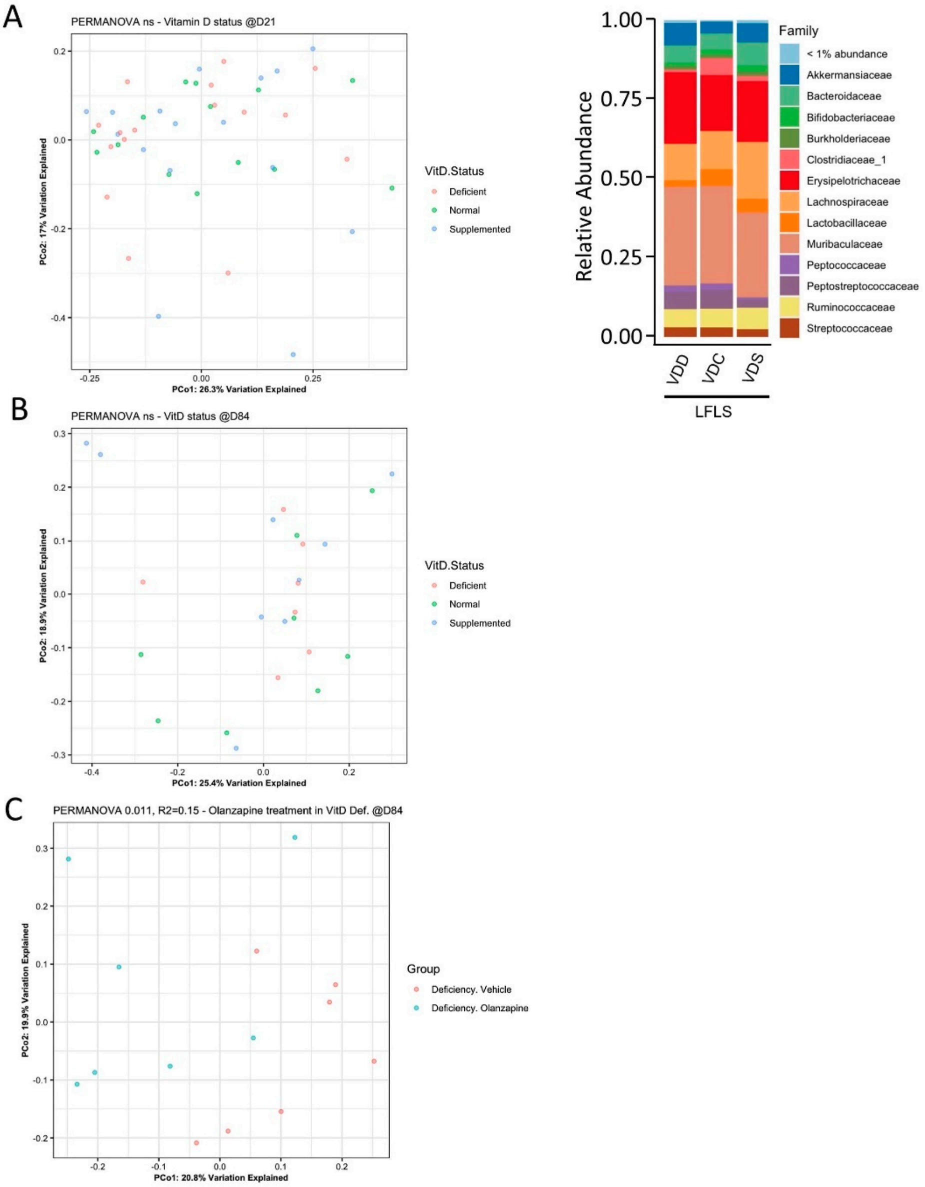

FigureS5: Relative abundance of bacterial taxa in after 3 weeks of LFLS VDD, VDC and VDS diets

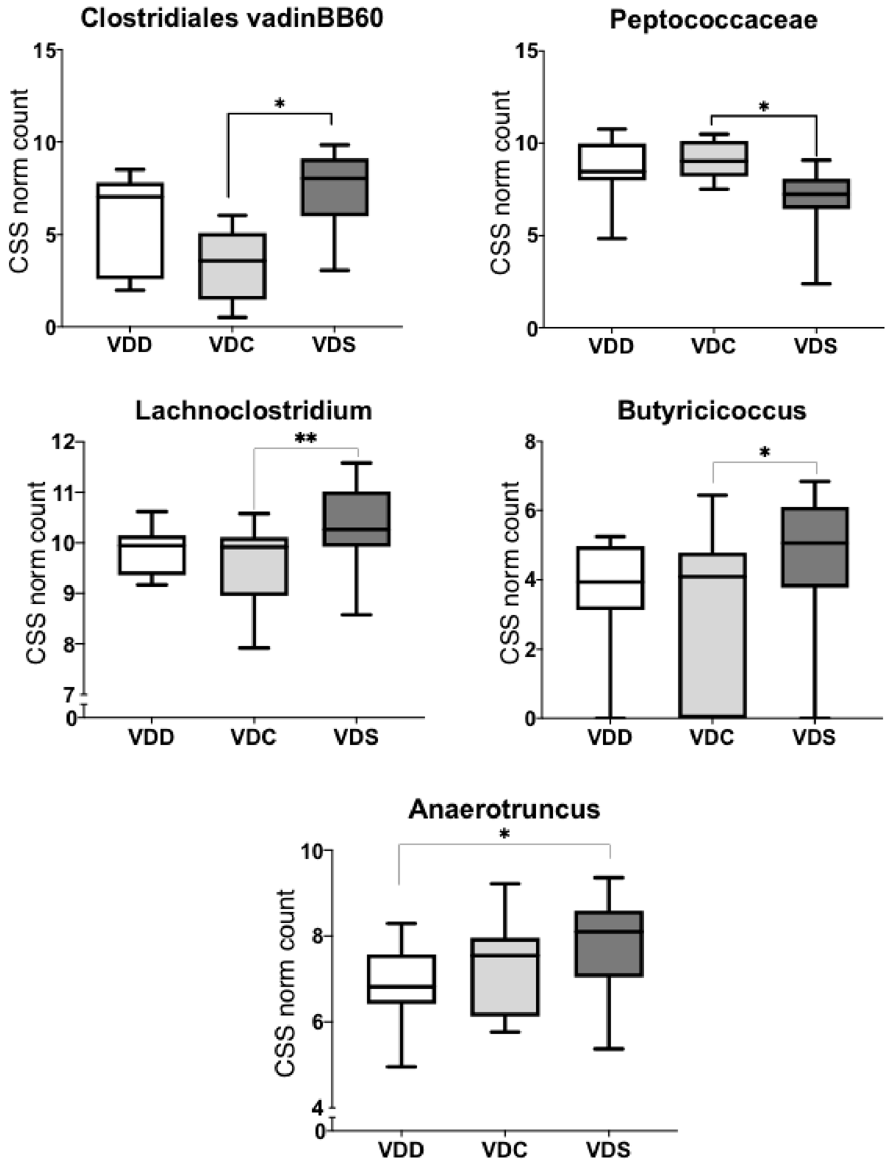

Supplement: Supplementary file 1 [file ijms-22-12361-s001.zip › ijms-1431912-supplementary.pdf]
